# A Correlational Study of Hearing Loss and Severity of Diabetic Retinopathy Among Jordanian Patients

**DOI:** 10.7759/cureus.43800

**Published:** 2023-08-20

**Authors:** Shehab A Al-Abed, Mai M Hakooz, Marwa H Teimat, Ghayda A Aldurgham, Wardeh K Alhusban, Anees A Hjazeen

**Affiliations:** 1 Department of Ophthalmology, Jordanian Royal Medical Services, Amman, JOR; 2 Department of Otolaryngology, Jordanian Royal Medical Services, Amman, JOR; 3 Department of Community Health Nursing, Jordanian Royal Medical Services, Amman, JOR

**Keywords:** risk factors, logistic regression, correlational and cross-sectional study, sensorineural hearing loss, diabetic retinopathy severity

## Abstract

Background

Sensorineural hearing loss (SNHL) is a common complication of diabetes, but the underlying mechanisms and risk factors are not fully understood. This study investigated the relationship between several potential risk factors and SNHL in 118 individuals previously diagnosed with diabetes mellitus type 2 (DM2).

Methods

We did a cross-sectional analysis of data collected from 118 patients with diabetes mellitus at multiple tertiary hospitals in the Jordanian Royal Medical Services (JRMS). The mean age was 60.57 ± 12.8 years, with 56.8% males. Demographic and clinical data were collected, including age, gender, duration of diabetes, presence and duration of hypertension, diabetic retinopathy (DR) severity, tinnitus, and comprehension inability. In addition, a hearing assessment for SNHL was performed using pure-tone audiometry at 0.5, 1, 2, 4, and 8 kHz frequencies. Goodman and Kruskal’s γ test and cumulative odds ordinal logistic regression were used to analyze the relationship between these factors and hearing assessment results for SNHL.

Results

Goodman and Kruskal’s γ test showed a statistically significant moderate and positive association between diabetic retinopathy severity and sensorineural hearing loss. Further analysis using logistic regression revealed that retinopathy severity, age, and gender were all significant predictors of hearing loss level in this population. The odds of having a higher level of hearing loss increased by 9.1% for each additional year of age and were higher for males than females. In terms of DR severity, a worsening SNHL had lower odds in individuals with no DR or mild nonproliferative DR compared to those with proliferative DR.

Conclusion

Our findings have important implications for managing SNHL in individuals with diabetes. They indicate that healthcare providers must closely monitor and manage the risk factors in patients with diabetes. Further research is needed to generalize better the relationship between these risk factors and SNHL, including the potential role of other factors, such as exposure to loud noise or certain medications.

## Introduction

Hearing loss is a common condition that affects individuals of all ages and can significantly impact their quality of life. It is estimated that hearing loss currently affects more than 1.5 billion people or 20% of the global population, with approximately 430 million of them having moderate or higher levels of hearing loss in the better hearing ear, and this number is expected to rise to over 700 million by 2050 [1]. Hearing loss is classified into conductive, sensorineural, or mixed-type hearing loss. One of the leading causes of sensorineural hearing loss (SNHL) is chronic exposure to loud noise. Still, other contributing factors include genetic predisposition, certain medications, and medical conditions such as diabetes mellitus (DM) and hypertension (HTN).

Diabetes mellitus is a chronic condition characterized by high blood sugar levels, which can lead to complications such as diabetic retinopathy (DR) and neuropathy. DR is a condition in which the blood vessels in the retina are damaged, leading directly or indirectly to vision loss. At the same time, neuropathy is nerve damage that can lead to sensory and motor deficits. Previous research has shown that individuals with DM are at an increased risk of developing SNHL [2]. Hypertension, or high blood pressure, is another common medical condition that can increase the risk of SNHL. High blood pressure can damage the inner ear's blood vessels and nerve fibers, leading to SNHL [3]. Also, hypertension is a risk factor for developing DR [4].

Given the potential relationship between DM, HTN, and SNHL, it is crucial to investigate the association between these conditions. This study examined the relationship between retinopathy severity, DM duration, HTN status, and duration, along with other factors like age, gender, and patient hearing-related symptoms and SNHL in a sample of individuals with DM. It was hypothesized that individuals with more severe retinopathy, a longer duration of DM, and a history of HTN would have worse SNHL.

## Materials and methods

Study design

This study was a cross-sectional analysis of data collected from patients having DM at multiple tertiary hospitals in the Jordanian Royal Medical Services (King Hussein Military Hospital, Amman; Prince Ali Bin Al-Hussein Military Hospital, Karak and Prince Zaid Bin Al-Hussein Military Hospital, Tafilah). The data was collected from two time periods, from April to August 2018 and from March to December 2022. Data collection started after obtaining Institutional Review Board (IRB) approval from the ethical committee at Jordanian Royal Medical Services. We assigned a case number to each patient so that no personal identifiers were used in the data for statistical analysis. All included patients signed a written statement of informed consent. The study was conducted in accordance with the Declaration of Helsinki.

Study participants

A total of 118 patients with DM were included in the study. Inclusion criteria were as follows: age 18 years or above, diagnosis of DM based on the American Diabetes Association criteria, and presence of retinopathy (any stage as graded according to the Early Treatment Diabetic Retinopathy Study scale) [5,6]. Exclusion criteria were a history of ear surgery, use of hearing aids, and presence of documented other causes of hearing loss.

Data collection

Recruitment was done through multiple avenues, including referrals from primary care physicians and internists within the medical center and patients attending their appointments for diabetic retinopathy examination checks in the ophthalmology specialty clinics. An ophthalmology specialist did a thorough history taking and anterior and posterior eye examination and then filled in the demographic and clinical data, and examination results as follows: age, gender, duration of DM, presence of HTN, duration of HTN, retinopathy severity (no DR, mild nonproliferative DR, or NPDR, moderate NPDR, severe NPDR, proliferative DR, or PDR), maculopathy status (no maculopathy, maculopathy, clinically significant macular edema, or CSME), symptoms of decreased hearing, inability to comprehend speech at normal speaking levels, and tinnitus.

Patients were then sent to do a hearing assessment for SNHL by a trained audiologist in a soundproof room using a calibrated audiometer. Pure-tone audiometry was performed at frequencies of 0.5, 1, 2, 4, and 8 kHz. The average hearing loss for each ear was calculated by summing the hearing thresholds at 500, 1000, 2000, 4000, and 8000 Hz, and then dividing the result by 5. SNHL was classified as normal, mild, moderate, or severe based on the World Health Organization criteria [1]. Ears with conductive and mixed hearing loss were excluded. Eyes with retinal vein or artery occlusions were excluded. The patients were labeled, first, with the eye with the worse retinopathy severity and maculopathy status, and second, with their worse ear’s hearing assessment.

Statistical analysis

Data from the two periods were combined and were analyzed using SPSS Statistics, version 28.0 (IBM Corp., Armonk, NY). Descriptive statistics were used to summarize the demographic and clinical characteristics of the study population. The relationship between retinopathy severity and hearing assessment for SNHL was assessed using Goodman and Kruskal’s γ test. Cumulative odds ordinal logistic regression was used to determine the independent predictors of hearing assessment for SNHL. A p-value of <0.05 was considered statistically significant, and a confidence interval of 95% was used.

## Results

This study included 118 individuals with type 2 diabetes. The mean age was 60.6±12.8 years, and the mean duration of diabetes was 13.9±8.8 years. In addition, 58.5% of the sample had a history of hypertension, and the mean duration of hypertension was 6.96 years. In terms of gender, the sample was somewhat evenly split, with 56.8% males and 43.2% females.

**Figure 1:**
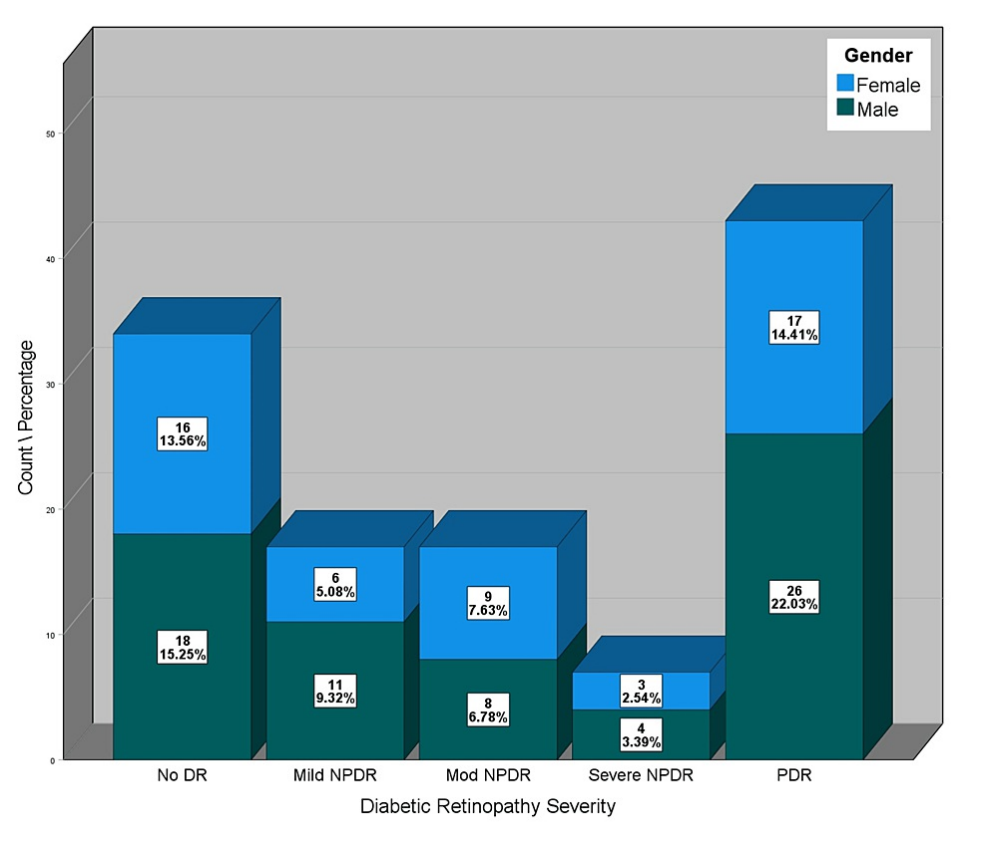
Distribution of diabetic retinopathy severity by gender DR: diabetic retinopathy; NPDR: nonproliferative DR; PDR: proliferative DR

The majority of the sample (77.1%) did not report symptoms of decreased hearing, while 22.9% reported having symptoms. Similarly, 79.7% did not report any symptom of an inability to comprehend, while 20.3% did. On the other hand, a relatively large proportion of the sample (41.5%) reported tinnitus symptoms. Considering retinopathy severity, 28.8% of the sample had no diabetic retinopathy, 14.4% had mild nonproliferative DR, 14.4% had moderate NPDR, 5.9% had severe NPDR, and 36.4% had proliferative DR. Figure 1 shows the distribution of DR along with gender.

Moreover, 37.3% of the sample had no maculopathy, 57.6% had maculopathy, and 5.1% had clinically significant macular edema at the time of examination.

**Figure 2:**
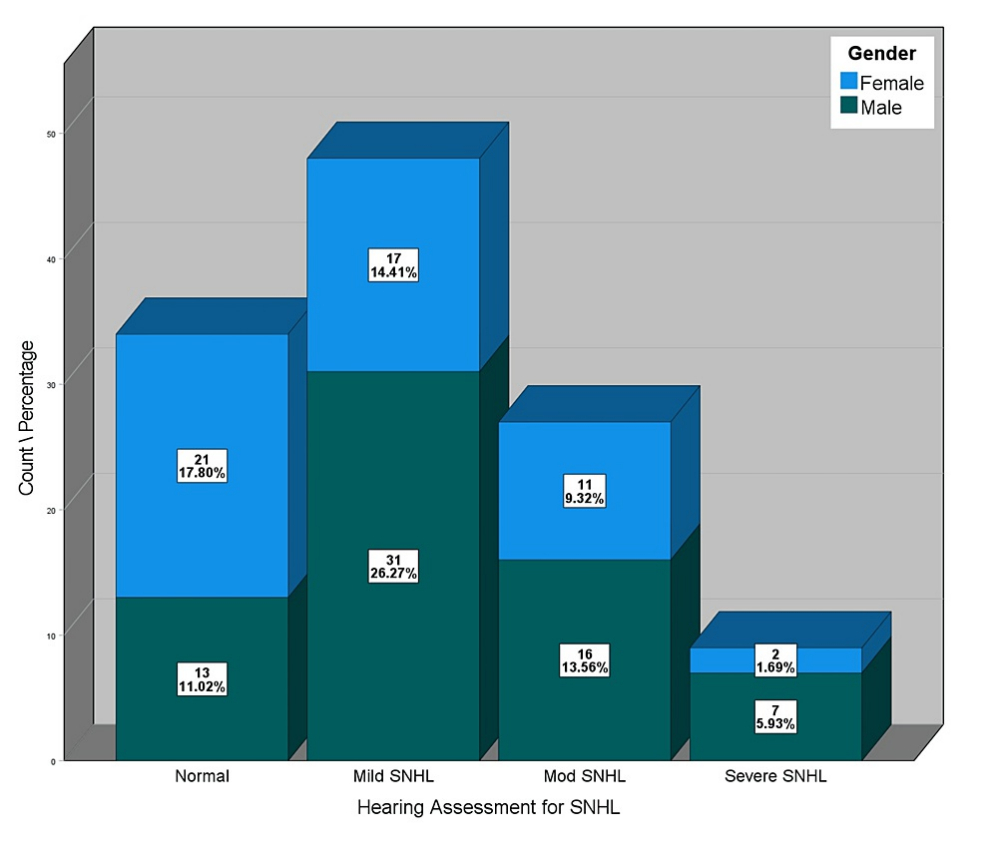
Distribution of hearing assessment for SNHL by gender SNHL: sensorineural hearing loss

The hearing assessment for sensorineural hearing loss revealed that 28.8% of the sample had normal hearing, 40.7% had mild SNHL, 22.9% had moderate SNHL, and 7.6% had severe SNHL. Figure 2 shows the distribution of SNHL by gender.

**Figure 3:**
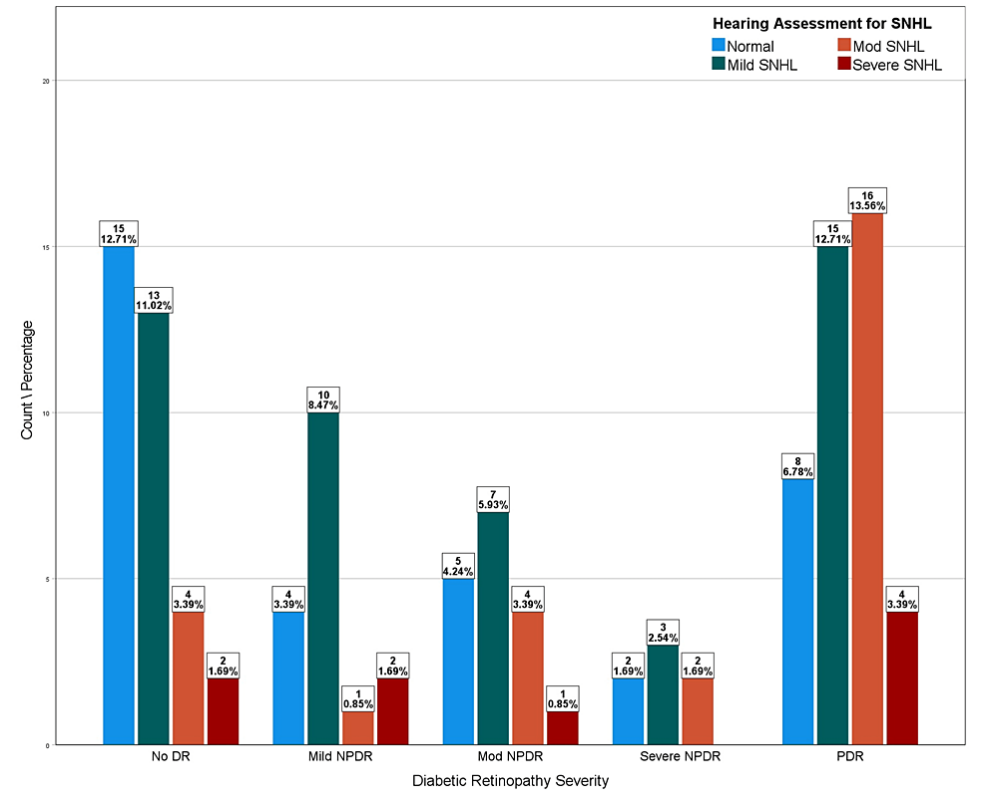
Distribution of SNHL assessment with diabetic retinopathy severity SNHL: sensorineural hearing loss; DR: diabetic retinopathy; NPDR: nonproliferative DR; PDR: proliferative DR

Table 1 shows the distribution of diabetic retinopathy with SNHL, and their relationship is further visualized in Figure 3.

**Table 1:** Association between diabetic retinopathy severity and SNHL assessment SNHL: sensorineural hearing loss; DR: diabetic retinopathy, NPDR: nonproliferative DR; PDR: proliferative DR

		Hearing assessment for SNHL				Total
		Normal	Mild SNHL	Moderate SNHL	Severe SNHL	
Retinopathy severity	No DR	15	13	4	2	34
	Mild NPDR	4	10	1	2	17
	Moderate NPDR	5	7	4	1	17
	Severe NPDR	2	3	2	0	7
	PDR	8	15	16	4	43
Total		34	48	27	9	118

When grouping the diabetic retinopathy as “no DR and mild NPDR” and “moderate NPDR, severe NPDR, and PDR” and calculating the odds ratio to have moderate or severe SNHL versus milder forms of SNHL, the odds ratio was 1.725.

Goodman and Kruskal’s γ was used to determine the association between diabetic retinopathy severity level (from “no DR” to “PDR”) and sensorineural hearing loss level (from “normal test” to “severe SNHL”). It showed a moderate, positive association that was statistically significant (G = 0.320, p = 0.002). An omnibus test was conducted to evaluate the model's cumulative odds ordinal logistic regression overall fit. The likelihood ratio chi-square was 108.992 (df = 13, p = .000), indicating that the model fit the data well.

Table 2 shows the tests of model effects used to assess each predictor’s contributions to the model.

**Table 2:** Association of diabetic retinopathy severity, clinical factors, and demographics with sensorineural hearing loss assessment ^a^Set to zero because this parameter is redundant. ^b^Fixed at the displayed value. B: unstandardized beta (coefficient); CSME: clinically significant macular edema; df: degrees of freedom; DM: diabetes mellitus; DR: diabetic retinopathy; Exp(B): odds ratio; HTN: hypertension; NPDR: nonproliferative DR; PDR: proliferative DR; Sig.: significance level; SNHL: sensorineural hearing loss; SE: standard error

Parameter		B	SE	95% Wald's confidence interval		Hypothesis test			Exp(B)	95% Wald's confidence interval for Exp(B)	
				Lower	Upper	Wald's chi-square	df	Sig.		Lower	Upper
Threshold	0 – normal test	3.464	1.6421	0.245	6.682	4.449	1	0.035	31.93	1.278	797.854
	1 – mild SNHL	6.739	1.7549	3.3	10.179	14.748	1	< .001	845.118	27.111	26344.862
	2 – moderate SNHL	10.056	1.9243	6.285	13.828	27.309	1	< .001	23299.826	536.247	1012373.1
0 – no DR		-2.115	0.8785	-3.837	-0.393	5.795	1	0.016	0.121	0.022	0.675
1 – mild NPDR		-1.795	0.7452	-3.256	-0.335	5.804	1	0.016	0.166	0.039	0.716
2 – moderate NPDR		-1.067	0.6458	-2.332	0.199	2.727	1	0.099	0.344	0.097	1.22
3 – severe NPDR		-1.955	0.9195	-3.757	-0.153	4.52	1	0.033	0.142	0.023	0.858
4 – PDR		0^a^	.	.	.	.	.	.	1	.	.
Female		-1.426	0.4419	-2.292	-0.56	10.415	1	0.001	0.24	0.101	0.571
Male		0^a^	.	.	.	.	.	.	1	.	.
0 – HTN		0.289	0.4464	-0.586	1.164	0.418	1	0.518	1.335	0.556	3.202
1 – no HTN		0^a^	.	.	.	.	.	.	1	.	.
0 – no maculopathy		0.305	1.1488	-1.946	2.557	0.071	1	0.791	1.357	0.143	12.894
1 – maculopathy		0.733	0.8995	-1.03	2.496	0.664	1	0.415	2.081	0.357	12.133
2 – CSME		0^a^	.	.	.	.	.	.	1	.	.
0 – decreased hearing		0.746	0.786	-0.794	2.287	0.901	1	0.342	2.109	0.452	9.842
1 – no decreased hearing		0^a^	.	.	.	.	.	.	1	.	.
0 – inability to comprehend		2.39	0.8496	0.725	4.056	7.915	1	0.005	10.917	2.065	57.716
1 – no inability to comprehend		0^a^	.	.	.	.	.	.	1	.	.
0 – tinnitus		1.265	0.5233	0.239	2.29	5.84	1	0.016	3.542	1.27	9.879
1 – no tinnitus		0^a^	.	.	.	.	.	.	1	.	.
Age		0.087	0.0234	0.041	0.133	13.917	1	< .001	1.091	1.042	1.142
DM duration		-0.008	0.0286	-0.064	0.048	0.08	1	0.777	0.992	0.938	1.049
Scale		1^b^									

The Wald chi-square for retinopathy severity was 9.410 (df = 4, p = .052), indicating a significant relationship between retinopathy severity and the level of hearing loss.

The odds of experiencing a worse sensorineural hearing loss compared to patients with proliferative diabetic retinopathy varied based on retinopathy severity. For patients with no diabetic retinopathy, the odds of worse SNHL were 0.121 (95% CI: 0.022 to 0.675), with a chi-square value of 5.795. Among individuals with mild NPDR, the odds increased to 0.166 (95% CI: 0.039 to 0.716), corresponding to a chi-square value of 5.804. In the case of moderate NPDR, the odds of worse SNHL were 0.344 (95% CI: 0.097 to 1.220), with a chi-square value of 2.727. For those with severe NPDR, the odds stood at 0.142 (95% CI: 0.023 to 0.858), and the associated chi-square value was 4.520.

The Wald chi-square for age was 13.917 (df = 1, p < .001), indicating a significant relationship between age and hearing loss level with an odds ratio of 1.091, 95% CI 1.042 to 1.142; χ^2^(1) = 13.917, p < .001. The estimate for the effect of age on hearing loss level was .087, indicating that for each additional year of age, the odds of having a higher hearing loss increased by 9.1%. The Wald chi-square for gender was 10.415 (df = 1, p = .001), indicating a significant relationship between gender and hearing loss level, where females were found to have a lower risk of severe hearing loss compared to males by an odds ratio of 0.240, 95% CI 0.101 to 0.571.

The odds of observing worse hearing test results were 10.917 times higher (95% CI: 2.065 to 57.716) among patients who reported the symptom of inability to comprehend compared to those who did not report this symptom. This effect reached statistical significance, as indicated by χ^2^(1) = 7.915, p = 0.005. Similarly, the odds of encountering worse hearing test results were 3.542 times greater (95% CI: 1.270 to 9.879) for patients with tinnitus than those without this symptom. This association was statistically significant with a χ^2^(1) = 5.840, p = 0.016.

The presence of HTN (χ^2^(1) = 0.418, p = 0.518), DM duration (χ^2^(1) = 0.080, p = 0.777), maculopathy stage (χ^2^(2) = 0.960, p = 0.619), and symptoms of decreased hearing (χ^2^(1) = 0.901, p = 0.342) were not significant predictors of hearing loss level.

## Discussion

The prevalence of diabetes in 1990 was 158.8 million, increasing to 459.9 million patients in 2019, constituting 6.18% of the world population [7]. It is a metabolic disorder with significant health impact as it increases the risk of heart attacks and strokes by two to three times [8], is among the leading causes of kidney failure [9], and causes diabetic retinopathy, which is a significant cause of blindness as a result of the proliferation of the retinal vessels [10].

Other morbidities might also be linked to diabetes, such as sensorineural hearing loss. Multiple studies showed that diabetic individuals were likelier to have SNHL than age- and sex-matched non-diabetics [11]. The exact mechanism by which diabetes may lead to SNHL is not well understood. However, it is thought that the microvascular changes and inflammation associated with this metabolic disorder may also affect the auditory system resulting in cochlear microangiopathy, degeneration of the stria vascularis, and loss of cochlear outer hair cells, which is hypothesized to be the cause behind SNHL associated with DM [12]. As microvascular changes cause diabetic retinopathy and are also hypothesized to cause sensorineural hearing loss, this study aimed to explore the relationship between the severity of diabetic retinopathy and the presence of and severity of sensorineural hearing loss.

Our results demonstrated that retinopathy severity was significantly associated with hearing assessment for sensorineural hearing loss. In particular, individuals who had more advanced retinopathy were found to have higher odds (odds ratio 1.725) of having poorer hearing assessment scores. This was evident among patients with moderate non-proliferative diabetic retinopathy or more severe retinopathy, who were more likely to experience moderate SNHL or worse. This finding is consistent with the previous research on the relationship between diabetic retinopathy and hearing loss [13,14].

Following the results in the previous section, a significant relationship between age and hearing loss level was found. The Wald chi-square statistic of 13.917 (df = 1, p < .001) indicated that this relationship was strong, with an odds ratio of 1.091. This relationship was further supported by the estimate for the effect of age on the hearing loss level, which indicated that the odds of having a higher level of hearing loss increased by 9.1% for each additional year of age. The 95% confidence interval of 1.042 to 1.142 further supports the reliability of these results. This finding aligns with previous research that found a strong relationship between age and hearing loss. A systematic review and meta-analysis by Lin et al. found that the prevalence of hearing loss increases with every decade of age [15]. Similarly, another study by Wasano et al. found that the prevalence of hearing loss increased significantly with age, with the highest prevalence occurring in individuals over the age of 80 [16]. These findings highlight the importance of regular hearing screenings and hearing protective measures in older individuals to prevent or mitigate the effects of age-related hearing loss.

Considering the gender of the patients and its relation to their hearing assessment scores, the study results showed a significant relationship, as indicated by the Wald chi-square statistic of 10.415 (df = 1, p = .001). This suggests that gender is a strong predictor of hearing loss level. Furthermore, females were found to have a lower risk of severe hearing loss compared to males, as indicated by the odds ratio of 0.240. This means that the odds of females having severe hearing loss are approximately 76% lower than the odds of males having severe hearing loss. The 95% confidence interval of 0.101 to 0.571 further supports the reliability of these results. This finding is supported by a previous research conducted in the mid-1990s that found that males are more than twice more likely to experience hearing loss compared to females [17], as opposed to a more recent study that found that this gender-related hearing loss difference is less than that reported before; they postulated that this decrement was due to changes in lifestyle and environmental circumstances [18].

It is worth noting that this study did not show a significant relationship between the presence of HTN as a comorbidity and SNHL severity (χ^2^(1) = 0.418, p = 0.518). However, when the study was split into hypertensive and non-hypertensive groups, the relationship between the HTN duration and the severity of SNHL was found to be significant with a Wald chi-square value of 7.129 (df = 1, p < .008) and an odds ratio of 1.136 and 95% CI 1.035 to 1.248. The estimate for the effect of duration (in years) on hearing loss level was 0.128, indicating that for each additional year of age, the odds of having a higher level of hearing loss increased by 12.8%. This might suggest that hypertension may be a risk factor for hearing loss. Furthermore, as the duration of HTN increases, the severity of hearing loss (as measured by the hearing assessment for SNHL) also tends to increase. This finding is consistent with a previous research that found a relationship between hypertension and hearing loss [3].

The duration of diabetes was calculated from the year of diagnosis, which might underestimate the actual number of years the patient had diabetes. Nevertheless, in the multinomial cumulative logit model, there was a significant relationship between this calculated DM duration and DR severity (χ^2^(1) = 38.295, p < 0.001, Exp(B)= 1.164) in the study group. Furthermore, the relationship between DM duration and SNHL severity was not statistically significant, with a significance of χ^2^(1) = 0.080, p = 0.777. A literature review showed different and inconsistent results; some studies found a positive correlation between DM duration and SNHL severity [19,20], whereas others found no significant relationship [21].

Patients were assessed for the presence and severity of diabetic maculopathy and were grouped into three groups: no diabetic maculopathy, diabetic maculopathy, and clinically significant macular edema. The provided data showed no significant relationship with hearing loss severity (χ^2^(2) = 0.960, p = 0.619).

We gathered information about hearing loss symptoms from patients. This data was collected through questions, in a yes/no format, asking about symptoms like tinnitus, decreased hearing, and inability to comprehend. Data analysis showed that two of these symptoms were significantly associated with hearing assessment for SNHL. In particular, the inability to comprehend had an odds ratio of 10.917, 95% CI 2.065 to 57.716 (χ^2^(1) = 7.915, p = .005), and patients with tinnitus were more likely to have the severe form of SNHL by 3.542 times (95% CI 1.270 to 9.879) as compared with patients not complaining of tinnitus (χ^2^(1) = 5.840, p = .016). This suggests that individuals who reported experiencing these symptoms were more likely to have SNHL. On the other hand, decreased hearing was not significantly associated with SNHL (χ^2^(1) = 0.901, p = 0.342). It is important to note that these symptoms may be a consequence of SNHL rather than a contributing factor. Still, the results of this study highlight the importance of considering the presence of symptoms in the evaluation and management of SNHL in diabetic patients.

It is worth noting that the present study had several limitations. First, the sample size was relatively small, which may have limited the power of the study to detect associations between variables. Additionally, using some self-report measures may aid in inaccurately capturing specific characteristics or experiences that are difficult to quantify or describe. Finally, the study was cross-sectional, so it is impossible to establish a causal relationship between retinopathy severity and hearing assessment for SNHL. Longitudinal studies will be needed to confirm the present study’s findings and further explore the potential mechanisms underlying the relationship between DR and hearing loss. Despite these limitations, the present study provides important insights into the relationship between DR and hearing loss. Moreover, further research is needed to understand this relationship and develop interventions to prevent or mitigate hearing loss in individuals with DR.

## Conclusions

In conclusion, the results of this study provide important insights into the relationship between several variables and hearing assessment for sensorineural hearing loss. The data analysis revealed that diabetic retinopathy severity, age, and male gender were significantly associated with worse SNHL; this finding is essential to consider when evaluating and managing SNHL in individuals with diabetes. Further research is needed to fully understand the mechanisms behind the relationship between diabetes and hearing loss and to develop effective strategies for preventing and mitigating hearing loss in individuals with diabetes.
